# Neuroprotective role of carvacrol in ischemic brain injury: a systematic review of preclinical evidence and proposed TRPM7 involvement

**DOI:** 10.3389/fphar.2025.1687119

**Published:** 2025-11-21

**Authors:** Abdulrahman M. Khojah, Hussain Al Dera

**Affiliations:** 1 College of Medicine, King Saud Bin Abdulaziz University for Health Sciences, Riyadh, Saudi Arabia; 2 King Abdullah International Medical Research Center (KAIMRC), Riyadh, Saudi Arabia

**Keywords:** carvacrol, TRPM7, ischemic stroke, global cerebral ischemia, neuroprotection, oxidative stress, apoptosis

## Abstract

**Introduction:**

Carvacrol, a phenolic monoterpene and putative TRPM7 inhibitor, has demonstrated neuroprotective activity in cerebral ischemia models. This systematic review synthesizes preclinical evidence on carvacrol across focal and global ischemia, separating outcomes by disease model and summarizing mechanisms of action and translational barriers.

**Methods:**

Following the Preferred Reporting Items for Systematic Reviews and Meta-Analyses guidelines, PubMed, ScienceDirect, and Google Scholar were searched from inception to 06-20-2025. Inclusion criteria (PICOS) targeted rodent models of focal (MCAO or hypoxia–ischemia) or global ischemia (BCCAO), with carvacrol administered systemically or centrally and reporting infarct size, behavior, oxidative stress, apoptosis, or pathway markers. Dual, independent screening, extraction, and SYRCLE risk-of-bias appraisal were performed. A prospectively registered protocol (KAIMRC NRR25/050/9) guided this review.

**Results:**

Seven studies met criteria. In focal ischemia (3 studies), individual experiments reported reduced infarct volume (up to 44%) and improved neurological scores when carvacrol was given before or shortly after injury. In global ischemia (4 studies), carvacrol improved memory/behavioral outcomes and neuronal survival in hippocampal CA1, with mixed effects on infarct surrogates. Across models, studies showed reduced oxidative damage (MDA, 4-HNE), increased antioxidant enzymes (SOD, CAT, GPx), lower apoptosis (cleaved caspase-3), and variable changes in TRPM7 expression. No study directly linked carvacrol’s outcome benefits to contemporaneous measurement of TRPM7 channel activity *in vivo*.

**Conclusion:**

Carvacrol demonstrates promising neuroprotective signals in both focal and global ischemia models, with convergent antioxidant and anti-apoptotic effects and suggestive TRPM7 involvement. However, evidence certainty is limited by small study numbers, heterogeneity, and methodological risks. Translation will require optimization of delivery, improved study design aligned with STAIR, and mechanistic validation using selective TRPM7 modulators.

**Systematic Review Registration:**

identifier NRR25/050/9.

## 1 Introduction

Stroke is an episode of acute neurological dysfunction caused by focal or global cerebral, spinal, or retinal vascular injury owing to hemorrhage or infarction, lasting beyond 24 h or leading to death with no apparent cause other than vascular damage (Sacco et al., 2013). Ischemic stroke, accounting for approximately 87% of all strokes, occurs following blood-flow disruption to the brain, inducing various pathological changes, including necrosis and neuroinflammation in neural tissues (Tsao et al., 2023; Li et al., 2024). Ischemic brain injury arises from either focal arterial occlusion (ischemic stroke) or global perfusion failure (cardiac arrest–like states) (Salaudeen et al., 2024). Although these entities share downstream pathways (ionic overload, mitochondrial dysfunction, oxidative stress, inflammation, apoptosis), they differ in onset dynamics, tissue vulnerability, and clinical course (Kuriakose and Xiao, 2020; Qin et al., 2022). Molecular and cellular mechanisms, such as oxidative stress, glial activation, and apoptosis, are crucial factors that influence the extent of neurological deficit (Salaudeen et al., 2024; Kuriakose and Xiao, 2020; Qin et al., 2022). Various methodologies, including ligation, laser embolization, and hypoxic chambers, have been used to simulate ischemic stroke conditions in preclinical settings (Singh et al., 2022; Amado et al., 2022). Depending on the research aim and the type of ischemic stroke, specific brain areas are targeted to replicate different stroke scenarios (Singh et al., 2022; Amado et al., 2022).

The melastatin-like transient receptor potential (TRPM) family comprises eight multifunctional cation channels (TRPM1–8) (Nadezhdin et al., 2023). Among them, TRPM7 is notable for its unique chanzyme structure, incorporating an ion channel and an intrinsic α-kinase domain at its C-terminus (Nadezhdin et al., 2023; Lin and Xiong, 2017). This structure allows TRPM7 to regulate ionic homeostasis and downstream signaling pathways (Lin and Xiong, 2017). In ischemia, TRPM7 acts as a master amplifier of neuronal injury (Bae and Sun, 2011). TRPM7, a divalent cation-permeable chanzyme, is potentiated by acidosis, ATP depletion, and reactive oxygen species and contributes to Ca^2+^/Zn^2+^ influx and downstream neurotoxicity (Bae and Sun, 2011; Jiang et al., 2005; Monteilh-Zoller et al., 2003; Gottschalk et al., 2018). The channel is potentiated by key pathological conditions, including extracellular acidosis (a pH of 6.0 increases current by 1.5–2 times) (Bae and Sun, 2011), reactive oxygen species (ROS) (Monteilh-Zoller et al., 2003), and ATP depletion (Gottschalk et al., 2018). Upon activation, TRPM7 facilitates a pronounced cation influx, particularly for Zn^2+^ (for which its pore is four times more permeable than for Ca^2+^) and Ca^2+^ (Jiang et al., 2005; Monteilh-Zoller et al., 2003). This ionic dysregulation triggers multiple destructive cascades, including Zn^2+^ neurotoxicity, Ca^2+^-dependent enzymatic damage, and disruption of the blood-brain barrier (BBB) via matrix metalloproteinase-9 activation (Sun et al., 2009; Alagawany et al., 2015; Mączka et al., 2023). Given its central role in mediating neuronal death, TRPM7 inhibition has become a promising neuroprotective strategy (Lin and Xiong, 2017; Jiang et al., 2005).

Carvacrol (5-isopropyl-2-methylphenol) is a naturally occurring phenolic monoterpenoid with established antioxidant, anti-inflammatory, and anesthetic properties (Abbasloo et al., 2023; Parnas et al., 2009). Natural products such as carvacrol can cross the blood-brain barrier and have been reported to inhibit TRPM7 *in vitro* (Zotti et al., 2013; Huet et al., 2017; Hong et al., 2018). Carvacrol is an inhibitor of the TRPM7 channel, with a reported half-maximal inhibitory concentration (IC_50_) of approximately 300 μM (Zotti et al., 2013). Studies have confirmed that carvacrol inhibits TRPM7 upregulation in the hippocampal CA1–CA3 regions of mammalian cells (Huet et al., 2017; Yu et al., 2012). This systematic review aims to compile the existing preclinical evidence on the efficacy of carvacrol in ischemic stroke models, focusing on its neuroprotective mechanisms, dose-response relationships, and the barriers to its clinical translation.

## 2 Materials and methods

### 2.1 Protocol and reporting

This systematic review followed a prospectively approved protocol (KAIMRC NRR25/050/9) and adhered to the Preferred Reporting Items for Systematic Reviews and Meta-Analyses (PRISMA) guidelines.

### 2.2 Search strategy

We conducted a comprehensive search on PubMed, ScienceDirect, and Google Scholar for studies published up to June 2025 (search period: 06-01-2025 to 06-20-2025). The search strategy combined keywords and Medical Subject Headings including: (carvacrol) AND (TRPM7 OR “transient receptor potential melastatin 7” OR TRPM) AND (stroke OR ischemia OR “global cerebral ischemia” OR BCCAO OR MCAO OR “hypoxia-ischemia”). Searches were restricted to English language publications, with no other filters applied. Reference lists of included studies were manually screened to identify additional relevant articles.

### 2.3 PICOS framework

- Population: Rodent models (rats, mice, gerbils); any sex; neonatal to adult; focal or global cerebral ischemia
- Intervention: Carvacrol, any dose/route/timing
- Comparator: Vehicle or standard control
- Outcomes: Infarct volume/neuronal loss; behavioral/neurological tests; oxidative stress markers; apoptosis/cell-survival markers; pathway readouts (e.g., TRPM7 expression)
- Study design: Controlled *in vivo* studies; relevant *in vitro* mechanistic data associated with *in vivo* models

### 2.4 Study selection and data extraction

Two reviewers (AK, HD) independently screened titles/abstracts and full texts to identify eligible studies, with discrepancies resolved by consensus. Studies were included if they (1) were original empirical research with quantitative outcomes on behavioral deficits, infarct volume, oxidative stress, or histological/molecular markers of cell death/viability; (2) involved carvacrol *in vivo* or *in vitro* in ischemic stroke models; (3) focused on brain-related outcomes; (4) considered established ischemia-induction methods (e.g., middle cerebral artery occlusion (MCAO), bilateral common carotid artery occlusion (BCCAO), or hypoxia-ischemia). Excluded studies were those with genetic TRPM7 modifications, multiple drugs targeting the same receptor, single-arm designs, or review articles.

**FIGURE 1:**
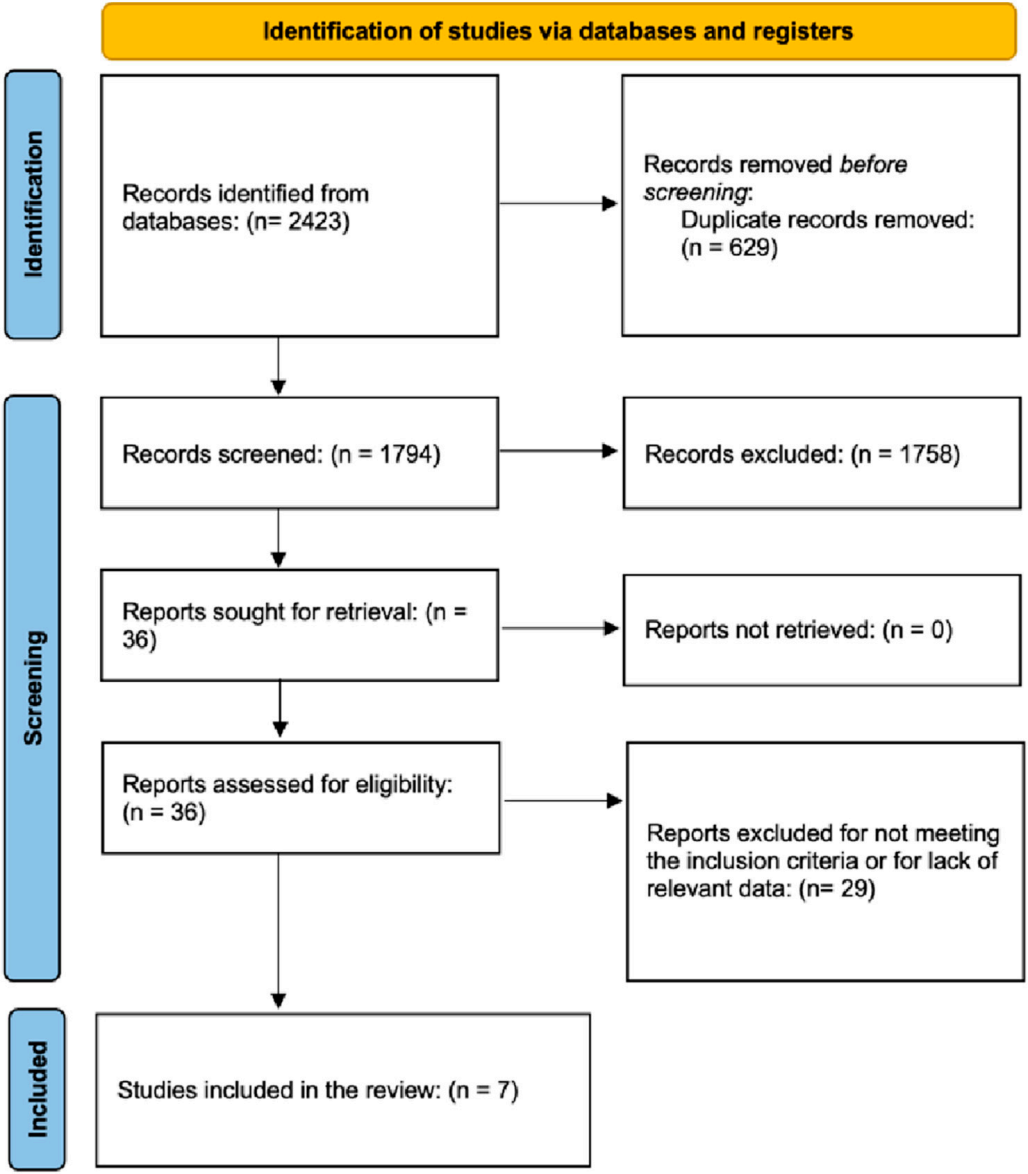
PRISMA flow diagram.

**FIGURE 2:**
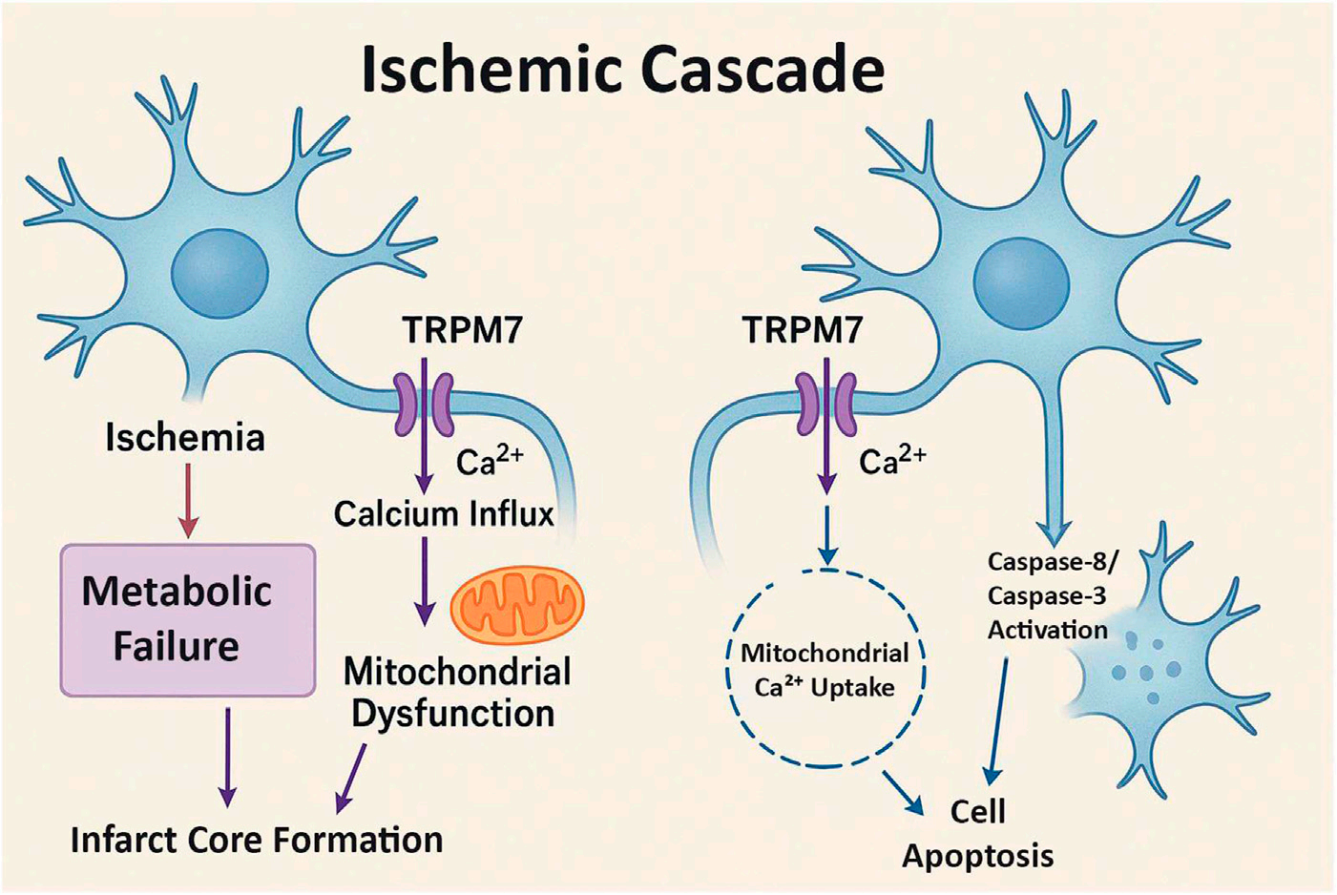
TRPM7-Mediated Pathways in Ischemic Stroke Injury. Schematic representation of TRPM7 channel activation and downstream pathways in ischemic stroke. During ischemia, TRPM7 channels facilitate calcium (Ca^2+^) influx, leading to two distinct pathways: (1) Left pathway - focal ischemia cascade involving mitochondrial dysfunction and subsequent focal ischemia cascade, and (2) Right pathway - direct caspase activation leading to cell apoptosis. Both pathways converge on neuronal cell death through different mechanisms but share the common upstream trigger of TRPM7-mediated calcium overload.

Data extraction was performed independently by two reviewers using a standardized spreadsheet archived with the protocol. Extracted variables included study characteristics, dosing, timing, routes, model type, and outcomes including study title, publication year, geographic region, animal model, ischemia induction method, injury duration, carvacrol dose and administration route, treatment frequency, evaluation time points, and outcomes (brain infarct size, behavioral impairment, cell death/viability markers, and oxidative stress indicators, including antioxidant levels). Data were compared between experimental and control groups to assess the effects of carvacrol. The study selection process is detailed in a PRISMA flow diagram (Figure 1). Adobe Photoshop, Eraser.io, perplexity.ai were used for the illustrations (Figure 2).

### 2.5 Risk of bias assessment

The included studies were evaluated for methodological quality using SYRCLE’s Risk of Bias tool, tailored for preclinical animal studies. Two reviewers independently applied SYRCLE’s tool. Domains assessed included sequence generation, allocation concealment, random housing, blinding of interventions and outcomes, random outcome assessment, incomplete data, selective reporting, baseline characteristics, and other bias (e.g., funding/conflicts, statistical methods, baseline imbalance, selective analysis). Discrepancies were resolved through discussion.

## 3 Results

### 3.1 Study selection, characteristics, and quality assessment

A comprehensive database search revealed 2,423 articles. After removing 629 duplicates, 1,794 unique records were screened by title and abstract, resulting in 36 articles selected for full-text reviews. Following the application of the inclusion criteria, 29 studies were excluded, leaving seven studies for qualitative synthesis (Yu et al., 2012; Chen et al., 2015; Başaran et al., 2022; Guan et al., 2019; Shahrokhi Raeini et al., 2020; Li et al., 2016; Aslam et al., 2025). These studies, published between 2012 and 2022, were conducted in China, Turkey, Iran, Korea, and Canada, among others, and involved rodent models, predominantly Sprague-Dawley and Wistar rats, ICR mice, and gerbils. Ischemic stroke was induced using the MCAO, BCCAO, or hypoxia-ischemia model, with or without reperfusion. Carvacrol was administered at doses typically ranging 25–100 mg/kg primarily via intraperitoneal (i.p.) or oral route, with timing varying from pre-insult to early post-insult administration. Multi-day treatment courses were employed in some global ischemia studies. The assessed outcomes included infarct volume, neurological deficit scores, oxidative stress markers, and neuronal apoptosis. The key characteristics of the included studies are summarized in Table 1.

**TABLE 1:** Baseline characteristics of included studies.

Author	Year	Location	Animal	Stroke method	Injury duration	Therapy type	Timing of administrations	Carvacrol dose/route
Basaran et al. (2022)	2022	Turkey	Wistar Rats	BCCAO/r	15 min	Post-injury	>24 h post-injury for 15 days	50 mg/kg (oral)
Guan et al. (2019)	2019	China	Gerbils	BCCAO/r	5 min	Post-injury	2 consecutive weeks	Not specified
Shahrokhi Raeini et al. (2020)	2019	Iran	Wistar Rats	BCCAO with 1-week interval	Permanent	Post-injury	From 3rd day after 2nd ligation to 8th week	Not specified
Hong et al. (2018)	2018	Korea	Sprague–Dawley Rats	BCCAO/r	7 min	Post-injury	Immediately after injury and daily for 3 days	50 mg/kg (i.p.)
Li et al. (2016)	2016	China	Sprague–Dawley Rats	MCAO/r	120 min	Post-injury	1 & 12 h post-injury	50 mg/kg (i.p.)
Chen et al. (2015)	2015	Canada	Sprague–Dawley Neonates	Right-CCAO and hypoxia	Permanent, 100-min hypoxia	Pre-injury	30 min pre-injury	10–50 mg/kg (i.p.)
Yu et al. (2012)	2012	China	ICR Mice	MCAO/r	75 min	Pre- and post-injury	2-h prior, during, and 2, 4, 6, and 7 h post-injury	25–50 mg/kg (i.p.)

*Legend*: BCCAO/r: Bilateral Common Carotid Artery Occlusion and Reperfusion; CCAO: Common carotid artery occlusion; MCAO/r: Middle Cerebral Artery Occlusion and Reperfusion; i.p.: intraperitoneal.

### 3.2 Effects on infarct volume and behavioral deficits by disease model

#### 3.2.1 Focal ischemia (MCAO and hypoxia-ischemia)

Three studies investigated the effects of carvacrol on infarct volume and behavioral deficits in focal ischemic stroke models, consistently demonstrating neuroprotective effects across various species and stroke models (Table 2). Yu et al. demonstrated a 44% reduction in infarct volume (p < 0.01) and improved neurological deficit scores 24 h post-injury in ICR mice subjected to 75-min MCAO when treated pre- and post-injury. Infarct volume decreases of up to ∼44% at 24 h were observed in mice when carvacrol was dosed pre-/peri-insult. Chen et al. found that the pre-injury administration of carvacrol in neonatal Sprague-Dawley rats subjected to hypoxia-ischemia significantly improved performance in the geotaxis reflex, cliff avoidance, and grip tests on Days 1, 3, and 7, alongside reduced infarct size at 24 h and on Day 7. Neonatal hypoxia-ischemia models showed infarct reductions at both 24 h and 7 days post-injury. Neurological function assessments revealed improved composite scores at 24 h, while neonatal sensorimotor tests demonstrated improvements across days 1–7.

**TABLE 2:** Studies of focal vs. global ischemia models: behavioral deficit and infarct size outcomes.

Author	Behavioral deficit	Assessment time points	Significantly improved assessment time points	Infarct size	Assessment timepoints	Significant improvement
(A) Focal ischemia models (MCAO/hypoxia-ischemia)						
Chen et al. (2015)	Significant improvement in geotaxis reflex, cliff avoidance, grip test	Days 1, 3, 7	1st, 3rd, 7th day in grip test; 3rd, 7th day in cliff avoidance; 7th day in geotaxis reflex	Significantly decreased	24 h and 7 days	24 h and 7 days
Yu et al. (2012)	Significant improvement in modified neurological deficit test	24 h post-injury	24 h post-injury	Significantly decreased (44% reduction)	24 h post-injury	24 h post-injury
Li et al. (2016)	Not assessed	N/A	N/A	Not directly measured	N/A	N/A

Author	Behavioral deficit	Assessment time points	Significantly improved assessment time points	Infarct size	Assessment timepoints	Significant improvement
(B) Global ischemia models (BCCAO).						
Guan et al. (2019)	Significant improvement in MWM	After 2-week treatment	After 2-week treatment	N/A	N/A	N/A
Shahrokhi Raeini et al. (2020)	Significant improvement in MWM	1-4th day and 6th day of Week 8	3rd, 4th day in escape latency; 4th day in traveled distance; 6th day in target quadrant time	N/A	N/A	N/A
Hong et al. (2018)	Not assessed	N/A	N/A	Indirectly assessed via neuronal death	3 days	3 days post-injury
Başaran et al. (2022)	Not assessed	N/A	N/A	N/A	N/A	N/A

Summary of evidence quality, study numbers, confidence levels, and clinical translation barriers for TRPM7 pathway components. Data compiled from systematic review of preclinical studies investigating carvacrol and TRPM7 inhibition in ischemic stroke models. Components are categorized by their role in the pathway (upstream targets, downstream consequences, therapeutic interventions) and their relevance to focal versus global ischemia models.

*Legend*: MWM: morris water maze test; N/A: not assessed.

#### 3.2.2 Global ischemia (BCCAO)

Four studies examined global ischemic models using bilateral common carotid artery occlusion (Table 2). Guan et al. reported significant improvements in the Morris Water Maze (MWM) performance in gerbils following 2 weeks of post-injury carvacrol treatment after 5-min BCCAO. Similarly, Raeini et al. observed enhanced MWM outcomes, including reduced escape latency and increased time spent in the target quadrant, in Wistar rats with permanent BCCAO treated between Day 3 and Week 8 post-injury. Behavioral assessments consistently showed improvements in Morris Water Maze performance after multi-day dosing regimens. Enhanced neuronal survival in hippocampal CA1 was demonstrated by histological analyses including Nissl staining, NeuN immunostaining, and TUNEL assays. Unlike focal ischemia models, outcomes in global ischemia studies often focused on hippocampal neuron viability rather than traditional infarct volume measurements, with mixed results for infarct surrogates.

### 3.3 Multimodal neuroprotective mechanisms

#### 3.3.1 TRPM7 inhibition and downstream effects

Two studies explored the role of carvacrol in modulating TRPM7 channels, which are implicated in neuronal death during ischemia. Hong et al. demonstrated that carvacrol (50 mg/kg, i.p.) significantly reduced TRPM7 expression by approximately 29% (p < 0.05) in the hippocampal CA1 region of Sprague-Dawley rats following BCCAO treatment, attenuating Zn^2+^ and Ca^2+^ influx and downstream neuroinflammatory pathways, including nuclear factor kappa B activation. Similarly, Chen et al. reported the suppression of TRPM7 in neonatal rats, which correlated with decreased neuronal damage. Studies that have reported reduced TRPM7 expression with carvacrol treatment didn’t directly report statistical causality between TRPM7 channel activity and the outcomes described within the same experiment.

#### 3.3.2 Mitigation of oxidative stress and apoptosis

All seven studies elucidated the antioxidant and anti-apoptotic properties of carvacrol, demonstrating consistent reductions in oxidative damage and apoptosis markers across models (Table 3; Figure 2). Basaran et al. reported a reduction in oxidative damage through elevated levels of superoxide dismutase (SOD), catalase (CAT), glutathione, and glutathione peroxidase in Wistar rats. Guan et al. and Raeini et al. observed increased activity of antioxidant enzymes (SOD and CAT) and decreased malondialdehyde (MDA) levels in the hippocampal CA1 region of gerbils and rats, respectively. Hong et al. noted diminished oxidative damage, as revealed by 4-hydroxynonenal (4-HNE) staining. Li et al. found elevated SOD levels and reduced MDA and inflammatory markers in cortical neurons. Additionally, Chen et al. and Yu et al. reported decreased cleaved caspase-3 activation and increased Bcl-2/Bax ratios, contributing to enhanced viability of cortical and hippocampal neurons. Oxidative stress markers consistently showed lower MDA and 4-HNE levels, while antioxidant enzymes including SOD, CAT, and glutathione peroxidase (GPx) were elevated. Reduced inflammatory markers were observed in some studies (Table 3).

**TABLE 3:** Studies on *in vitro* models of cell death and viability, oxidative stress, and carvacrol dose–response relationship.

Author	Cell death and viability	Indication	Cell type investigated	Oxidative stress	Indication	Cell type investigated	Dose dependency	Indication
Başaran et al. (2022)	N/A	N/A	N/A	Significant decrease in oxidative damage and significant protection through antioxidant mechanisms	SOD, CAT, GSH, GSH-Px, and TBARS levels	Not-Specified	NO	N/A
Guan et al. (2019)	Significant increase in cell survival and viability, along with a significant decrease in cell death	Nissl staining, NeuN Immunohistochemistry, TUNEL immunofluorescence, APP, and GPx4	Hippocampal CA1 cells	Significant protection through antioxidant mechanisms	SOD, Mn-SOD, Cu/Zn-SOD, GSH-Px, CAT, and MDA	Hippocampal CA1 cells	YES	Behavioral deficit test
Shahrokhi Raeini et al. (2020)	Significant increase in healthy cell density	Nissl staining	Hippocampal CA1 cells	Significant protection through antioxidant mechanisms	MDA, SOD, CAT, and DPPH	Hippocampal CA1 cells	YES	Behavioral deficit test, DPPH, CAT
Hong et al. (2018)	Significant decrease in cell death	FJB staining	Hippocampal CA1, CA2, and hippocampal subiculum cells	Significant decrease in oxidative damage	4HNE staining	Hippocampal CA1, CA2, and hippocampal subiculum cells	NO	N/A
Li et al. (2016)	N/A	N/A	N/A	Significant protection through antioxidant mechanisms	SOD, MDA, COX-2 (mRNA expression), INOS (mRNA expression)	Cortical neurons	YES	Cytokines and MPO
Chen et al. (2015)	Significant increase in cell survival and viability, along with a significant decrease in cell death	PI fluorescence, NeuN, TUNEL immunofluorescence, Cleaved Caspase-3 Western blot, Bcl-2/Bax protein ratio	Cortical neurons	N/A	N/A	N/A	YES	Infarct volume and cell death
Yu et al. (2012)	Significant decrease in cell death	Cleaved Caspase-3 Western blot	Not-Specified	N/A	N/A	N/A	YES	Infarct volume

*Legend*: APP: amyloid precursor protein; FJB: Fluoro-Jade B; SOD: superoxide dismutase; CAT: catalase; GSH: glutathione; GSH-Px: Glutathione Peroxidase; TBARS: thiobarbituric acid reactive substances; MDA: malondialdehyde; MPO: myeloperoxidase; PI: propidium iodide; 4HNE: 4-Hydroxynonenal; COX-2: Cyclooxygenase-2; INOS: inducible nitric oxide synthase; DPPH: 2,2-Diphenyl-1-picrylhydrazyl.

#### 3.3.3 Modulation of pro-survival pathways

Three studies explored the activation of pro-survival pathways by carvacrol. Yu et al. demonstrated increased phosphorylation of Akt in mice, which was reversed by phosphoinositide 3-kinase (PI3K) inhibitors, indicating the involvement of the PI3K/Akt pathway. Furthermore, Guan et al. and Li et al. suggested that carvacrol activates the nuclear factor erythroid 2-related factor 2/heme oxygenase-1 (Nrf2/HO-1) pathway, leading to the upregulation of antioxidant gene expression. However, the direct evidence for this pathway in cerebral ischemia remains limited. Evidence of PI3K/Akt and Nrf2/HO-1 activation was observed in select studies, contributing to enhanced neuronal resilience following ischemic insult (Table 3; Figure 2).

### 3.4 Dose-response relationship and timing considerations

Five studies reported dose-dependent effects of carvacrol on neuroprotection (Table 3; Figure 2). Yu et al. found that 50 mg/kg was more effective than 25 mg/kg in reducing infarct volume in mice. Chen et al. noted superior infarct reduction at 50 mg/kg compared with that at 30 mg/kg in neonatal rats. Guan et al., Raeini et al., and Li et al. observed dose-dependent changes in antioxidant enzyme activities and inflammatory markers. Higher doses (e.g., 50 mg/kg) tended to outperform lower doses in individual studies. Efficacy appeared greatest with dosing before or within a few hours after ischemic onset for systemic delivery routes. One study demonstrated that intracerebroventricular administration extended the therapeutic window compared to systemic delivery (Table 3). However, optimal treatment regimens remain uncertain due to heterogeneity in experimental protocols across studies.

### 3.5 Risk of bias and quality assessment

The quality of evidence in each study was evaluated using SYRCLE’s Risk of Bias tool in Table 4. SYRCLE appraisal revealed frequent unclear sequence generation and allocation concealment, limited random housing protocols, and incomplete blinding procedures across the included studies.

**TABLE 4:** Risk of bias assessment using SYRCLE’s risk of bias tool.

Author	Sequence generation	Baseline characteristics	Allocation concealment	Random housing	Blinding (intervention)	Random outcome assessment	Blinding (outcome)	Incomplete outcome data	Selective outcome reporting	Other sources of bias
Başaran et al. (2022)	?	✓	✓	?	−	−	−	✓	✓	−
Guan et al. (2019)	?	✓	✓	?	✓	✓	✓	✓	✓	✓
Shahrokhi Raeini et al. (2020)	?	✓	✓	?	−	−	−	✓	✓	✓
Hong et al. (2018)	−	✓	−	?	✓	✓	✓	✓	✓	✓
Li et al. (2016)	?	✓	✓	?	−	−	−	✓	✓	✓
Chen et al. (2015)	?	✓	✓	✓	✓	✓	✓	✓	✓	✓
Yu et al. (2012)	?	✓	✓	?	−	−	−	✓	✓	✓

*Legend*: (✓) indicates low risk of bias; (−) indicates high risk of bias; (?) indicates unclear or missing information.

## 4 Discussion

### 4.1 Interpretation by disease model

Separating focal from global ischemia clarifies that behavioral and hippocampal survival benefits are more consistently reported in global ischemia studies, whereas infarct reduction and early neurological improvement are reported in focal ischemia (Hong et al., 2018; Yu et al., 2012; Başaran et al., 2022; Shahrokhi Raeini et al., 2020; Li et al., 2016). Shared antioxidant and antiapoptotic effects suggest convergent downstream actions despite model differences (Yu et al., 2012; Chen et al., 2015; Başaran et al., 2022).

### 4.2 Mechanistic appraisal

Carvacrol’s inhibition of TRPM7 is biologically plausible and supported by reduced expression in some studies, but definitive causality is unproven *in vivo* (Zotti et al., 2013; Yu et al., 2012). Off-target actions (e.g., TRPV3/TRPA1, GABA_A receptor modulation) and robust antioxidant effects could contribute independently (Melo et al., 2010; Kessler et al., 2014; Niu et al., 2022). Future work should incorporate selective TRPM7 modulators (e.g., NS8593) alongside outcome measures to establish mechanism (Chubanov et al., 2005; Chubanov and Gudermann, 2020; Chubanov et al., 2020). The specificity of carvacrol’s effect on TRPM7 remains unclear owing to its interactions with other molecular targets (Melo et al., 2010; Kessler et al., 2014; Niu et al., 2022; Earley et al., 2010; Talavera et al., 2009). Carvacrol activates TRPV3 and TRPA1 channels and modulates GABA_A receptors, which could contribute to its neuroprotective effects or introduce confounding off-target effects (Melo et al., 2010; Kessler et al., 2014; Niu et al., 2022; Earley et al., 2010; Talavera et al., 2009). For example, Melo et al. demonstrated carvacrol’s anxiolytic-like effects in mice, potentially via GABA_A receptor modulation, suggesting a broader pharmacological profile (Earley et al., 2010). In contrast, Khalil et al. found the neuroprotective effects of carvacrol in a status epilepticus model were not mediated through GABA_A receptors or sodium channels, emphasizing TRPM7 inhibition as the primary mechanism (Talavera et al., 2009). This discrepancy highlights the need for further studies to clarify whether GABAergic effects contribute significantly to carvacrol’s benefits in stroke models (Earley et al., 2010; Talavera et al., 2009). Additionally, the antioxidant properties of carvacrol, as evidenced by reduced malondialdehyde (MDA) levels and increased superoxide dismutase (SOD) and catalase (CAT) activities in the studies of Yu et al. and Al Dera et al., suggest a potential TRPM7-independent mechanism (Yu et al., 2012; Li et al., 2016). These antioxidant effects could directly mitigate oxidative stress, a key contributor to ischemic injury, without TRPM7 inhibition (Yu et al., 2012; Li et al., 2016).

### 4.3 Translational considerations

Key barriers include short half-life and bioavailability, off-target activity at higher concentrations, a narrow systemic therapeutic window, and methodological weaknesses in preclinical studies (Table 5) (Lin and Xiong, 2017; Abbasloo et al., 2023; Yu et al., 2012; Chen et al., 2015; Başaran et al., 2022; Guan et al., 2019; Shahrokhi Raeini et al., 2020; Li et al., 2016; Aslam et al., 2025). Adoption of STAIR-aligned practices (randomization, blinding, appropriate models, and *a priori* statistical plans) and use of embolic/photothrombotic models may improve predictive validity (Singh et al., 2022; Amado et al., 2022; Dong et al., 2012). Exploration of formulation strategies and structure-activity relationship (SAR)-guided analog development could improve selectivity and pharmacokinetics (Yu et al., 2012; Busey et al., 2023). Despite robust preclinical evidence showing carvacrol’s effects in reducing infarct volume and behavioral deficits, several barriers impede clinical translation (Lin and Xiong, 2017; Abbasloo et al., 2023; Yu et al., 2012; Chen et al., 2015; Başaran et al., 2022; Guan et al., 2019; Shahrokhi Raeini et al., 2020; Li et al., 2016; Aslam et al., 2025):

- Poor Pharmacokinetics: Carvacrol has a short plasma half-life (∼1.5–4.5 h in various animal models) due to rapid hepatic metabolism primarily by cytochrome P450 enzyme CYP2A6, limiting sustained therapeutic brain levels (Hong et al., 2018; Khalil et al., 2017).
- Lack of Target Specificity: At concentrations >100 μM, carvacrol interacts with multiple off-target receptors including TRPV3, TRPA1, and GABA_A receptors, which may cause unwanted effects such as sedation or altered neuronal excitability (Melo et al., 2010; Kessler et al., 2014; Niu et al., 2022; Earley et al., 2010; Talavera et al., 2009).
- Narrow Therapeutic Window: Neuroprotection is optimal when administered within 2–3 h post-stroke onset for systemic delivery; intracerebroventricular administration extends this window up to 6 h. This presents challenges in clinical settings where delayed presentation is common (Yu et al., 2012; Başaran et al., 2022).
- Limitations of Preclinical Models: Predominant use of ligation-based models like intraluminal filament middle cerebral artery occlusion (MCAO), which poorly replicate human thromboembolic stroke pathophysiology, may overestimate efficacy (Singh et al., 2022; Amado et al., 2022; Al Dera et al., 2022). Methodological flaws such as inadequate randomization and blinding increase bias risk (Dong et al., 2012).

**TABLE 5:** Barriers to clinical translation.

Barrier category	Specific issues	Impact level	Potential solutions
Pharmacokinetic	Short half-life (1.8–2 h), poor bioavailability, rapid metabolism	High	Nanoformulations, sustained-release systems, prodrug approaches
Pharmacodynamic	Off-target effects (TRPV3, TRPA1, GABA_A), unclear dose-response in humans	Moderate	SAR-guided analog development, selective TRPM7 modulators
Therapeutic Window	Narrow systemic window (2–3 h), timing-dependent efficacy	High	Improved delivery methods, combination therapies, extended dosing regimens
Mechanistic	Indirect evidence for TRPM7 involvement, multiple pathways activated	Moderate	Direct TRPM7 activity measurement, genetic validation studies
Preclinical Quality	Poor study design, lack of randomization/blinding, publication bias	High	STAIR-compliant protocols, multi-center validation, negative result publication
Species Translation	Young healthy animals vs. elderly patients with comorbidities	High	Aged animal models, comorbidity models, relevant stroke subtypes
Regulatory	Natural product variability, standardization issues, safety profile	Moderate	Synthetic analogs, standardized formulations, comprehensive toxicology
Commercial	Limited patent protection, low commercial interest in natural products	Low	Novel formulations, combination products, orphan drug pathways

*Legend*: Impact Level refers to the severity of the barrier for clinical translation (High = major obstacle, Moderate = significant challenge, Low = manageable issue.

### 4.4 Future directions and proposed solutions

To overcome these challenges and facilitate clinical application of carvacrol or TRPM7-targeted therapies, the following are recommended:

- Development of Selective TRPM7 Inhibitors: More potent and selective blockers like NS8593 (IC_50_ ∼1.6 μM) exhibit fewer off-target effects compared to carvacrol. Though direct evidence in stroke models is limited, their ability to modulate TRPM7 suggests promising neuroprotective potential warranting further investigation (Chubanov et al., 2005; Chubanov and Gudermann, 2020; Chubanov et al., 2020; Fisher et al., 2009; Busey et al., 2023).
- Improved Preclinical Study Design: Use of clinically relevant embolic or photothrombotic stroke models better mimics human stroke. Strict adherence to STAIR guidelines (randomization, blinding, and robust statistical analysis) will improve study reliability and translational relevance (Singh et al., 2022; Amado et al., 2022; Dong et al., 2012).
- Formulation and Analog Development: Optimization of delivery systems and synthesis of analogs guided by SAR analysis could enhance pharmacokinetic profile and target selectivity (Yu et al., 2012; Busey et al., 2023).

### 4.5 Limitations of this review

The evidence base is limited and heterogeneous, preventing meta-analysis (Yu et al., 2012; Chen et al., 2015; Başaran et al., 2022; Guan et al., 2019; Shahrokhi Raeini et al., 2020; Li et al., 2016; Aslam et al., 2025). Some studies lacked detailed methodology. Despite following a prospectively approved protocol, notable data sparsity impeded subgroup analyses.

## 5 Conclusion

Carvacrol shows neuroprotective signals across focal and global ischemia models, with consistent antioxidant and anti-apoptotic effects and suggestive TRPM7 involvement. However, evidence certainty is limited by small study numbers, heterogeneity, and methodological risks. Translation will require optimization of delivery, improved study design aligned with STAIR, and mechanistic validation using selective TRPM7 modulators. Stronger mechanistic validation and STAIR-compliant preclinical designs are needed before considering clinical translation. SAR-informed analogs and improved delivery strategies represent promising avenues.

The clinical translation of carvacrol is hindered by pharmacokinetic limitations, lack of specificity, a narrow therapeutic window, and reliance on suboptimal preclinical models. The controversies surrounding its mechanism of action, particularly the role of antioxidant and GABAergic effects, underscore the need for studies using selective TRPM7 blockers. By leveraging advanced drug delivery systems, developing selective inhibitors, improving preclinical study designs, and exploring biomarker-guided therapies, the therapeutic potential of carvacrol or TRPM7-targeted strategies can be fully realized, facilitating effective stroke treatments.

## Data Availability

The original contributions presented in the study are included in the article/supplementary material, further inquiries can be directed to the corresponding author.

## References

[B1] Abbasloo E. Khaksari M. Sanjari M. Kobeissy F. Thomas T. C. (2023). Carvacrol decreases blood-brain barrier permeability post-diffuse traumatic brain injury in rats. Sci. Rep. 13 (1), 14546. 10.1038/s41598-023-40915-x 37666857 PMC10477335

[B2] Al Dera H. Alassiri M. Kahtani R. A. Eleawa S. M. AlMulla M. K. Alamri A. (2022). Melatonin attenuates cerebral hypoperfusion-induced hippocampal damage and memory deficits in rats by suppressing TRPM7 channels. Saudi J. Biol. Sci. 29 (4), 2958–2968. 10.1016/j.sjbs.2022.01.018 35531206 PMC9073071

[B3] Alagawany M. El-Hack M. Farag M. R. Tiwari R. Dhama K. (2015). Biological effects and modes of action of carvacrol in animal and poultry production and Health—A review. Adv. Anim. Vet. Sci. 3 (2s), 73–84. 10.14737/journal.aavs/2015/3.2s.73.84

[B4] Amado B. Melo L. Pinto R. Lobo A. Barros P. Gomes J. R. (2022). Ischemic stroke, lessons from the past towards effective preclinical models. Biomedicines 10 (10), 2561. 10.3390/biomedicines10102561 36289822 PMC9599148

[B5] Aslam H. Albaqami F. Rehman N. U. Shah F. A. (2025). Carvacrol attenuated myocardial infarction through NLRP3-mediated pyroptosis and mTOR/Nrf2/PPARγ-dependent autophagic signaling. Toxicol. Appl. Pharmacol. 498, 117281. 10.1016/j.taap.2025.117281 40064378

[B6] Başaran M. Öztanır M. N. Çiftçi O. Türkmen N. B. Akyuva Y. Önder A. (2022). Neuroprotective effect of carvacrol in an experimental cerebral ischemia and reperfusion rat model. J. Clin. Exp. Med. 39 (2), 454–458. 10.52142/omujecm.39.2.28

[B46] Bae C. Y. Sun H. S. (2011). TRPM7 in cerebral ischemia and potential target for drug development in stroke. Acta Pharmacologica Sinica. 32 (6), 725–733. 21552293 10.1038/aps.2011.60 PMC4009967

[B8] Busey G. W. Manjegowda M. C. Huang T. Iobst W. H. Naphade S. S. Kennedy J. A. (2023). Novel TRPM7 inhibitors with potent anti-inflammatory effects in vivo. bioRxiv. 2023–05.

[B10] Chen W. Xu B. Xiao A. Liu L. Fang X. Liu R. (2015). TRPM7 inhibitor carvacrol protects brain from neonatal hypoxic-ischemic injury. Mol. Brain 8, 11. 10.1186/s13041-015-0102-5 25761704 PMC4337201

[B11] Chubanov V. Gudermann T. (2020). Mapping TRPM7 function by NS8593. Int. J. Mol. Sci. 21 (19), 7017. 10.3390/ijms21197017 32977698 PMC7582524

[B12] Chubanov V. Gudermann T. Schlingmann K. P. (2005). Essential role for TRPM6 in epithelial magnesium transport and body magnesium homeostasis. Pflugers Arch. 451 (1), 228–234. 10.1007/s00424-005-1470-y 16075242

[B45] Dong R. H. Fang Z. Z. Zhu L. L. Ge G. B. Cao Y. F. Li X. B. (2012). Identification of CYP isoforms involved in the metabolism of thymol and carvacrol in human liver microsomes (HLMs). Die Pharmazie—An Int. J. Pharm. Sci. 67 (12), 1002–1006. 23346763

[B13] Earley S. Gonzales A. L. Garcia Z. I. (2010). A dietary agonist of transient receptor potential cation channel V3 elicits endothelium-dependent vasodilation. Mol. Pharmacol. 77 (4), 612–620. 10.1124/mol.109.060715 20086034 PMC2845943

[B14] Fisher M. Feuerstein G. Howells D. W. Hurn P. D. Kent T. A. Savitz S. I. (2009). Update of the stroke therapy academic industry roundtable preclinical recommendations. Stroke 40 (6), 2244–2250. 10.1161/STROKEAHA.108.541128 19246690 PMC2888275

[B15] Gottschalk B. Richler A. Srour S. Becker I. C. Wolf K. Stritt S. (2018). TRPM7 kinase controls calcium responses in arterial thrombosis and stroke in mice. Arterioscler. Thromb. Vasc. Biol. 38 (2), 344–352. 10.1161/ATVBAHA.117.310391 29146750

[B16] Guan X. Li X. Yang X. Yan J. Shi P. Ba L. (2019). The neuroprotective effects of carvacrol on ischemia/reperfusion-induced hippocampal neuronal impairment by ferroptosis mitigation. Life Sci. 235, 116795. 10.1016/j.lfs.2019.116795 31470002

[B18] Hong D. K. Choi B. Y. Kho A. R. Lee S. H. Jeong J. H. Kang B. S. (2018). Carvacrol Attenuates hippocampal neuronal death after global cerebral ischemia *via* inhibition of transient receptor potential melastatin 7. Cells 7 (12), 231. 10.3390/cells7120231 30486272 PMC6315386

[B19] Huet S. Tago A. Kasus-Jacobi A. Martin G. (2017). Pharmacokinetic analysis of thymol, carvacrol and diallyl disulfide after intramammary and topical applications in goats. Vet. Res. Commun. 41 (2), 139–148. 10.1007/s11259-017-9681-8 28210926

[B20] Jiang J. Li M. Yue L. (2005). Potentiation of TRPM7 inward currents by protons. J. Gen. Physiol. 126 (2), 137–150. 10.1085/jgp.200409185 16009728 PMC2266571

[B21] Kessler A. Sahin-Nadeem H. Lummis S. C. (2014). GABAA receptor modulation by terpenoids from Sideritis extracts. Mol. Nutr. Food Res. 58 (4), 851–862. 24273211 10.1002/mnfr.201300420 PMC4384808

[B22] Khalil A. Kovac S. Morris G. Walker M. C. (2017). Carvacrol after status epilepticus (SE) prevents recurrent SE, early seizures, cell death, and cognitive decline. Epilepsia 58 (2), 263–273. 10.1111/epi.13645 28084627

[B23] Kuriakose D. Xiao Z. (2020). Pathophysiology and treatment of stroke: present status and future perspectives. Int. J. Mol. Sci. 21 (20), 7609. 10.3390/ijms21207609 33076218 PMC7589849

[B24] Li Z. Hua C. Pan X. Fu X. Wu W. (2016). Carvacrol exerts neuroprotective effects *via* suppression of the inflammatory response in Middle cerebral artery occlusion rats. Inflammation 39 (4), 1566–1572. 10.1007/s10753-016-0392-5 27324156

[B25] Li X. Y. Kong X. M. Yang C. H. Cheng Z. F. Lv J. J. Guo H. (2024). Global, regional, and national burden of ischemic stroke, 1990-2021: an analysis of data from the global burden of disease study 2021. EClinicalMedicine 75. 39157811 10.1016/j.eclinm.2024.102758 PMC11327951

[B26] Lin J. Xiong Z. G. (2017). TRPM7 is a unique target for therapeutic intervention of stroke. Int. J. Physiol. Pathophysiol. Pharmacol. 9 (6), 211–216. 29348798 PMC5770518

[B27] Mączka W. Twardawska M. Grabarczyk M. Wińska K. (2023). Carvacrol—A natural phenolic compound with antimicrobial properties. Antibiotics 12 (5), 824. 10.3390/antibiotics12050824 37237727 PMC10215463

[B28] Melo F. H. Venâncio E. T. de Sousa D. P. de França Fonteles M. M. de Vasconcelos S. M. M. Viana G. S. B. (2010). Anxiolytic-like effect of Carvacrol (5-isopropyl-2-methylphenol) in mice: involvement with GABAergic transmission. Fundam. Clin. Pharmacol. 24 (4), 437–443. 10.1111/j.1472-8206.2009.00788.x 19909350

[B29] Monteilh-Zoller M. K. Hermosura M. C. Nadler M. J. Scharenberg A. M. Penner R. Fleig A. (2003). TRPM7 provides an ion channel mechanism for cellular entry of trace metal ions. J. Gen. Physiol. 121 (1), 49–60. 10.1085/jgp.20028740 12508053 PMC2217320

[B30] Nadezhdin K. D. Correia L. Narangoda C. Patel D. S. Neuberger A. Gudermann T. (2023). Structural mechanisms of TRPM7 activation and inhibition. Nat. Commun. 14 (1), 2639. 10.1038/s41467-023-38362-3 37156763 PMC10167348

[B31] Niu C. Sun X. Hu F. Tang X. Wang, K. (2022). Molecular determinants for the chemical activation of the warmth-sensitive TRPV3 channel by the natural monoterpenoid carvacrol. J. Biol. Chem. 298 (3). 35150742 10.1016/j.jbc.2022.101706 PMC8920929

[B32] Parnas M. Peters M. Dadon D. Lev S. Vertkin I. Slutsky I. (2009). Carvacrol is a novel inhibitor of Drosophila TRPL and mammalian TRPM7 channels. Cell. Calcium 45 (3), 300–309. 10.1016/j.ceca.2008.11.009 19135721 PMC2680423

[B33] Qin C. Yang S. Chu Y. H. Zhang H. Pang X. W. Chen L. (2022). Signaling pathways involved in ischemic stroke: molecular mechanisms and therapeutic interventions. Signal Transduct. Target Ther. 7 (1), 215. 10.1038/s41392-022-01064-1 35794095 PMC9259607

[B34] Sacco R. L. Kasner S. E. Broderick J. P. Caplan L. R. Connors J. J. B. Culebras A. (2013). An updated definition of stroke for the 21st century: a statement for healthcare professionals from the American Heart Association/American Stroke Association. Stroke 44 (7), 2064–2089. 10.1161/STR.0b013e318296aeca 23652265 PMC11078537

[B35] Salaudeen M. A. Bello N. Danraka R. N. Ammani M. L. (2024). Understanding the pathophysiology of ischemic stroke: the basis of Current therapies and opportunity for new ones. Biomolecules 14 (3), 305. 10.3390/biom14030305 38540725 PMC10968326

[B36] Shahrokhi Raeini A. Hafizibarjin Z. Rezvani M. E. Safari F. Afkhami Aghda F. Zare Mehrjerdi F. (2020). Carvacrol suppresses learning and memory dysfunction and hippocampal damages caused by chronic cerebral hypoperfusion. Naunyn Schmiedeb. Arch. Pharmacol. 393 (4), 581–589. 10.1007/s00210-019-01754-8 31729545

[B37] Singh A. A. Kharwar A. Dandekar M. P. (2022). A review on preclinical models of ischemic stroke: insights into the pathomechanisms and new treatment strategies. Curr. Neuropharmacol. 20 (9), 1667–1686. 10.2174/1570159X19666210907092928 34493185 PMC9881062

[B38] Sun H. S. Jackson M. F. Martin L. J. Jansen K. Teves L. Cui H. (2009). Suppression of hippocampal TRPM7 protein prevents delayed neuronal death in brain ischemia. Nat. Neurosci. 12 (10), 1300–1307. 10.1038/nn.2395 19734892

[B39] Talavera K. Gees M. Karashima Y. Meseguer V. M. Vanoirbeek J. A. J. Damann N. (2009). Nicotine activates the chemosensory cation channel TRPA1. Nat. Neurosci. 12 (10), 1293–1299. 10.1038/nn.2379 19749751

[B40] Tsao C. W. Aday A. W. Almarzooq Z. I. Anderson C. A. Arora P. Avery C. L. (2023). Heart disease and stroke Statistics-2023 update: a report from the American heart Association. Circulation 147 (8), e93–e621. 10.1161/CIR.0000000000001123 36695182 PMC12135016

[B42] Yu H. Zhang Z. L. Chen J. Pei A. Hua F. Qian X. (2012). Carvacrol, a food-additive, provides neuroprotection on focal cerebral ischemia/reperfusion injury in mice. PLoS One 7 (3), e33584. 10.1371/journal.pone.0033584 22438954 PMC3306416

[B44] Zotti M. Colaianna M. Morgese M. G. Tucci P. Schiavone S. Avato P. (2013). Carvacrol: from ancient flavoring to neuromodulatory agent. Molecules 18 (6), 6161–6172. 10.3390/molecules18066161 23708230 PMC6270539

