# Correction: Neuroprotective role of carvacrol in ischemic brain injury: a systematic review of preclinical evidence and proposed TRPM7 involvement

**DOI:** 10.3389/fphar.2025.1751557

**Published:** 2025-12-08

**Authors:** Abdulrahman M. Khojah, Hussain Al Dera

**Affiliations:** 1 College of Medicine, King Saud Bin Abdulaziz University for Health Sciences, Riyadh, Saudi Arabia; 2 King Abdullah International Medical Research Center (KAIMRC), Riyadh, Saudi Arabia

**Keywords:** carvacrol, TRPM7, ischemic stroke, global cerebral ischemia, neuroprotection, oxidative stress, apoptosis

There was a mistake in Table 1 as published. The references added to the first column were inserted incorrectly. The corrected Table 1 appears below.

**TABLE 1:** Baseline characteristics of included studies.

Author	Year	Location	Animal	Stroke method	Injury duration	Therapy type	Timing of administrations	Carvacrol dose/route
Basaran et al. (2022)	2022	Turkey	Wistar rats	BCCAO/r	15 min	Post-injury	>24 h post-injury for 15 days	50 mg/kg (oral)
Guan et al. (2019)	2019	China	Gerbils	BCCAO/r	5 min	Post-injury	2 consecutive weeks	Not specified
Shahrokhi Raeini et al. (2020)	2019	Iran	Wistar rats	BCCAO with 1-week interval	Permanent	Post-injury	From 3rd day after 2nd ligation to 8th week	Not specified
Hong et al. (2018)	2018	Korea	Sprague–dawley rats	BCCAO/r	7 min	Post-injury	Immediately after injury and daily for 3 days	50 mg/kg (i.p.)
Li et al. (2016)	2016	China	Sprague–dawley rats	MCAO/r	120 min	Post-injury	1 and 12 h post-injury	50 mg/kg (i.p.)
Chen et al. (2015)	2015	Canada	Sprague–dawley neonates	Right-CCAO and hypoxia	Permanent, 100-min hypoxia	Pre-injury	30 min pre-injury	10–50 mg/kg (i.p.)
Yu et al. (2012)	2012	China	ICR mice	MCAO/r	75 min	Pre- and post-injury	2-h prior, during, and 2, 4, 6, and 7 h post-injury	25–50 mg/kg (i.p.)

BCCAO/r, Bilateral Common Carotid Artery Occlusion and Reperfusion; CCAO, common carotid artery occlusion; MCAO/r, Middle Cerebral Artery Occlusion and Reperfusion; i. p., intraperitoneal.

The original article has been updated.

